# Cleavage of Human Embryos: Options and Diversity

**Published:** 2016

**Authors:** Yu. K. Doronin, I. V. Senechkin, L. V. Hilkevich, M. A. Kurcer

**Affiliations:** Lomonosov Moscow State University, Faculty of Biology, Leninskie Gory, 1, bldg. 12, Moscow, 119991 , Russia; Perinatal Medical Center, Department of Infertility Treatment and IVF, Sevastopolskiy prosp., 24, bldg.1, Moscow, 117209, Russia

**Keywords:** human embryos, time-lapse analysis, cleavage, blastomere genealogy

## Abstract

In order to estimate the diversity of embryo cleavage relatives to embryo progress (blastocyst formation), time-lapse imaging data of preimplantation human embryo development were used. This retrospective study is focused on the topographic features and time parameters of the cleavages, with particular emphasis on the lengths of cleavage cycles and the genealogy of blastomeres in 2- to 8-cell human embryos. We have found that all 4-cell human embryos have four developmental variants that are based on the sequence of appearance and orientation of cleavage planes during embryo cleavage from 2 to 4 blastomeres. Each variant of cleavage shows a strong correlation with further developmental dynamics of the embryos (different cleavage cycle characteristics as well as lengths of blastomere cycles). An analysis of the sequence of human blastomere divisions allowed us to postulate that the effects of zygotic determinants are eliminated as a result of cleavage, and that, thereafter, blastomeres acquire the ability of own syntheses, regulation, polarization, formation of functional contacts, and, finally, of specific differentiation. This data on the early development of human embryos obtained using noninvasive methods complements and extend our understanding of the embryogenesis of eutherian mammals and may be applied in the practice of reproductive technologies.

## INTRODUCTION

In the past few decades, with the rapid development and commercialization of reproductive technologies, preimplantation human embryos have become the focus of close attention. Ethical norms and legal constraints have put limitations on all but non-invasive methods in the study of conceptus; i.e. only microscopic observations. As a result of comprehensive phenomenological research, a system for the assessment of the morphology and rate of development of early human embryos *in vitro *was developed which allows one to predict the appearance of implantation-competent (high-quality) blastocysts with a higher or lesser degree of certainty [1, 2]. The introduction of incubators equipped with a continuous video recording device into the practice of reproductive medicine has significantly expanded the possibilities of diagnosis and prognosis of embryo quality [3-5]. Currently, a large body of factual data has been accumulated in the archives of centers of human reproductive biology a thorough analysis of which can not only improve the prognostic capabilities of the morphokinetic approach to the identification of promising embryos, but also deepen our understanding of the early development of placental mammals.

The first (meridional) cleavage furrow of the polarized zygote of placental mammals begins at the animal pole (in the immediate vicinity of the polar body) and extends from the animal to the vegetal pole [6-10]. Cleavage furrows of the blastomeres of 2-cell embryos are orthogonal to the first cleavage furrow and distributed in planes approximately coinciding with the equatorial and meridional planes of the zygote. Thus, there are four possible variants of cleavage of 2-cell embryo blastomeres: the two blastomeres divide either in the equatorial (E) or meridional (M) direction (variants EE or MM) or the first division is meridional and the second is equatorial (variant ME) and vice versa (variant EM).

The listed combinations of second divisions of the cleavage have been described in mouse embryos, with the variants for 4-cell embryos being different in phenotype. In ME and EM divisions, blastomeres form a tetrahedral structure (i.e. they are projected onto the corners of an imaginary tetrahedron). As a result of EE or MM divisions, blastomeres are distributed in the form of a plate or rosette [11, 12]. According to canonical descriptions, a human 4-cell embryo, unlike mouse 4-cell embryos, is formed as a result of successive meridional and equatorial divisions of 2-cell embryo blastomeres and, thereafter, assumes a tetrahedral form [6, 10, 13]. At the same time, the existence of substantive differences, including those in the ways of 4-cell embryo formation, at such early (phylogenetically ancient) basic stages of the embryogenesis of laboratory mammals and humans seems improbable.

The appearance of variants of 4-cell embryos as a result of second divisions of the cleavage seems to be a significant event in early ontogenesis. The differences in ooplasmic segregation are associated with the differences in the dynamic pattern of subsequent embryo development [14, 15], which, on the contrary, would normally result in the formation of a homogenous final structure: i.e. a blastocyst. In other words, this phenomenon should be assumed as one of the reasons for the diversity in subsequent development and, thereafter, the earliest manifestation of the regulatory ability of mammalian embryos.

Based on the assumptions presented above, the purpose of this study is (1) to conduct a detailed analysis of the topographic features of the second division of the cleavage of cultured human embryos; and (2) to analyze the variations in the time parameters of subsequent stages of embryo cleavage that are different in the variants of the second cleavage. Comparison of the genealogy of blastomeres with the sequence of their divisions allowed us to assume the role of cleavage as predetermining the normal course of the events accompanying embryo compaction and cavitation.

## EXPERIMENTAL

The material used in the study was time-lapse video recordings of 101 human embryos (microscope Primo Vision incorporated into a Thermo thermostat; video capture every 15 min) obtained under the standard culturing protocol from 20 anonymous patients aged 27 to 44 years (average 34.7 years). The orientation of the first three cleavage divisions in relation to the animal- vegetal axis of the zygote and successive moments of the division of the zygote and each blastomere until the 16-cell stage of development were determined by viewing images of time-lapse recording of individual embryos. The genealogy of blastomeres was traced in parallel. Based on these registrations, 4-cell embryos were classified in accordance with the division variants of the second cycle of cleavage and the durations of the zygotic period (from fertilization to zygote division) and the cleavage cycles were estimated: i.e. the difference in the onset time of the third and first divisions of blastomeres (the second cycle of cleavage), the seventh and third divisions (the third cycle of cleavage), and the 15th and seventh divisions (the fourth cycle of cleavage). The duration of the periods not accompanied by cell division (difference in the onset time of the second and first, fourth and third, eighth and seventh divisions), and the periods of cell divisions (difference in the time of the third and second, seventh and fourth, 15th and eighth divisions) of the second, third, and fourth cleavage cycles were also calculated (see *Fig. 2*). These measurements allowed us to calculate the duration of the cycles of individual blastomeres as the difference between the division moments of corresponding mother and daughter blastomeres. Based on these data, one can reconstruct and compare the genealogy of blastomeres before the 16-cell stage using the duration of blastomere cycles as their marker. The results of the measurement were analyzed and compared using non-parametric methods of variation statistics on the Stadia (A.P. Kulaichev, Lomonosov Moscow State University) and Statistica v6 (StatSoft) software. At the end of the standard cultivation period, the stages of embryo development were diagnosed in accordance with the established classification [16, 17].

**Fig. 1:**
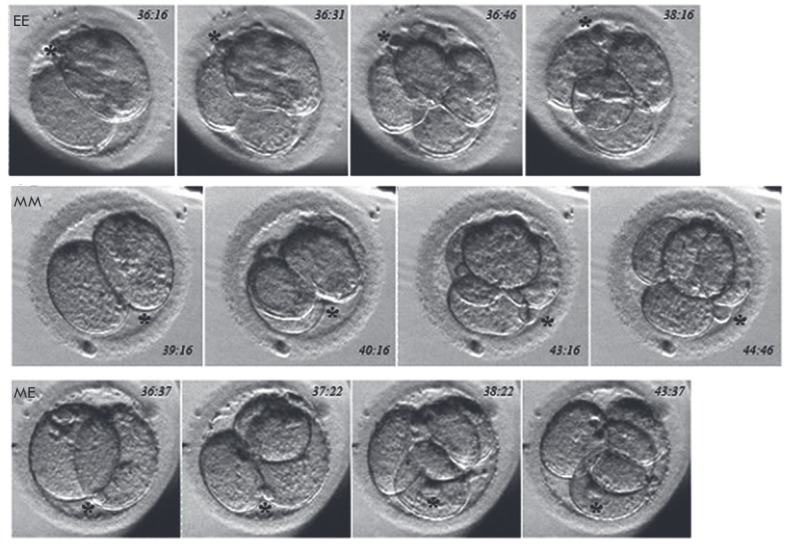
Examples of sequential equatorial (EE), meridional (MM), and meridional followed by equatorial (ME) blastomere divisions and final configuration of a 4-cell embryo in relation to 4-cell embryo formation. Equatorial followed by meridional (EM) cleavage type (not shown) is different from ME cleavage type only in the order of division. The time after intracellular sperm injection (h:m) is indicated on the figure. Asterisk depicts the position of the second polar body

Various developmental anomalies were noted by the first divisions of the cleavage of 33 embryos: excessive fragmentation, extremely short or long cell cycles (dozens of minutes or two days and more, and even complete absence of cytokinesis during the entire observation period), fusion of blastomeres right after division, and giant intracellular vacuoles and extracellular cavities in 4 to 8-cell embryos. These embryos were excluded from consideration. However, in some cases, measurable parameters of their first cycles were compared with the corresponding parameters in embryos that developed in the absence of organic disorders.

**Fig. 2:**
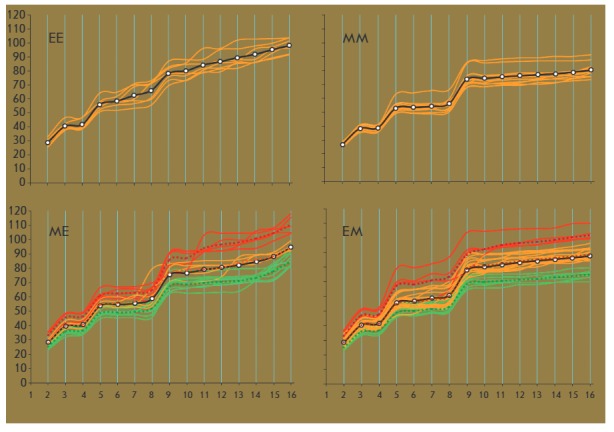
Embryo cleavage trajectories for EE, MM, ME, and EM cleavage type embryos. The green, red, and yellow lines indicate embryos with high, low, and medium cleavage intensities, respectively. Dashed lines indicate average embryo cleavage trajectories for each significantly different cleavage intensity group. Solid lines with markers depict average trajectories for each embryo variant. Horizontal axes stand for the number of blastomeres. Vertical axes indicate the time after intracellular sperm injection (h)

## RESULTS AND DISCUSSION

**Relationship between dividing blastomeres**

EE, MM, ME, and EM variants of blastomere divisions were detected in 10.3, 19.1, 27.9 and 42.6 % of all cases where 2-cell embryos had developed without structural abnormalities (N = 68). In ME or EM cases, two pairs of sister cells were oriented almost mutually perpendicular to each other due to the orthogonality of division planes for 2-cell embryo blastomeres, while a 4-cell embryo adopted a configuration close to a tetrahedron. Eventually, the geometric correctness of such a tetrahedron is improved due to the slow movements of sister pairs of blastomeres (*Fig. 1*). As a result of EE or MM divisions, a sort of plate or rosette of blastomeres is formed. In these cases, the sister pairs of blastomeres are shifted into a mutually perpendicular orientation. As a result, the plate or rosette also adopts a tetrahedron form (*Fig. 1*). Thus, the form of human 4-cell embryos becomes homogeneous regardless of the orientation of previous cleavage furrows. The displacement of cell pairs is most probably associated with the optimization of the form of 4-cell embryos as a result of the alignment of the mechanical stresses that take place in the limited volume of an embryo after blastomere divisions. Association of sister blastomeres is probably due to the long-term persistence of cytoplasmic bridges [8, 11, 18]. Long-term contact between sister blastomeres promotes the appearance of cell clusters–a compact arrangement of the descendants of 2-, 4- and 8-cell embryo blastomeres. We noticed the formation of such clusters upon reconstruction of blastomere genealogy.

The frequencies of appearance of EE, MM, ME, and EM variants in embryos with structural defects (30.3, 15.2, 24.2 and 30.3 %, respectively; N = 33) do not differ from the frequencies for embryos that developed without structural abnormalities (χ2 = 6.471, *P *= 0.091) but do not correspond to the distribution typical for the latter (χ2 = 15.130, *P *= 0.002). This is due to the fact that the proportions of MM, ME, and EM variants of the second cleavage in the two groups of embryos are identical (χ2 values for the corresponding alternative comparisons are 0.17, 0.09, 0.65; *P *= 0.682, 0.763 and 0.419, respectively). The division frequency in abnormal embryos has a clear tendency to outreach (almost three-fold) the frequency in normal embryos (χ2 = 4.30,* P *= 0.038; Yates’ correction *P *= 0.072). Probably, it should be assumed that EE embryos are to a greater extent prone to organic disturbances in development than embryos with other variants of second divisions of the cleavage. The same tendency was noted in mouse EE embryos [19].

**Cleavage cycles and trajectories**

Variants of blastomere divisions in the second cleavage cycle are essential for further development. A smoothed wave-like time trajectory of development is typical for EE embryos, while MM, ME, and EM embryos are characterized by a pronounced step-like trajectory (*Fig. 2*). Average time trajectories (see *Fig. 2*) differ from each other (paired Wilcoxon test; significance of the differences (*P*) between EE and MM, EE and EM, MM and ME, MM and EM variants equals 0.001; significance of the differences between the EE and EM variants is 0.002; and 0.023 between ME and EM variants).

Differences in the duration of cleavage cycles become apparent in the third cleavage cycle and increase in the 4th cycle. EE embryos have the longest cycles, while MM embryos have the shortest cycles, with EM and ME embryos occupying an intermediate position. Lengthening of the overall cycles of cleavage is mainly due to the extension of cell division periods. Therefore, the division frequency is maximal for MM and minimal for EE embryos (*Table 1*). MM embryos reach the 16-cell stage after 80.5 ± 4.85 hours (mean ± standard deviation); EM embryos – after 87.7 ± 9.47 h; and ME and EM embryos – after 94.8 ± 11.29 and 98.1 ± 5.05 h, respectively (the differences of the mean values are statistically significant except for the differences for the EE and ME groups (*P *= 0.197); Van der Waerden test).

**Table 1:** Time parameters of the cleavage cycles (mean value and standard deviation, h) of embryos with different variants of successive divisions of 2-cell embryo blastomeres

		EE	MM	ME	EM
Number and prospective stages of embryo development		B5(2), B4(1), B3(1), B1(3)	B5(7), B4(3), B3(2), B1(1)	B5(3), B4(5), B3(2), B2(2), B1(5), M(2)	B5(8), B4(6), B3(5), B2(4), B1(5), M(1)
Zygotic period		28.6 ± 2.70^1^	26.7 ± 1.44^1^	28.2 ± 3.96	28.5 ± 3.61
Overall cycle duration	cycle 2	13.1 ± 2.52	12.1 ± 1.05	12.4 ± 1.22	12.8 ± 1.85
	cycle 3	24.4 ± 5.72^2,3,4^	17.4 ± 3.33^2^	18.1 ± 5.58^3^	19.4 ± 4.96^4^
	cycle 4	32.1 ± 8.67^5,6^	24.3 ± 3.06^5, 7, 8^	36.0 ± 8.12^7, 9^	26.9 ± 4.57^6, 8, 9^
Period without divisions	cycle 2	11.9 ± 1.33	11.6 ± 1.12	11.5 ± 1.16	11.9 ± 1.48
	cycle 3	14.3 ± 2.81	14.1 ± 2.78	13.2 ± 1.88	14.3 ± 3.72
	cycle 4	12.1 ± 3.36^10,11^	17.5 ± 4.05^10^	16.8 ± 7.04	17.3 ± 4.67^11^
Period of blastomere divisions	cycle 2	1.1 ± 1.60	0.5 ± 0.31^12,13^	0.9 ± 0.55^12^	1.0 ± 0.89^13^
	cycle 3	10.0 ± 4.57^14,15,16^	3.3 ± 2.08^14,17^	4.9 ± 5.07^15^	5.1 ± 2.99^16,17^
	cycle 4	20.0 ± 6.87^18,19^	6.8 ± 2.47^18,20^	19.2 ± 5.93^20,21^	9.6 ± 5.29^19,21^
Mean time between successive cell divisions ^**^	cycle 2	0.6 ± 0.80	0.2 ± 0.15	0.4 ± 0.28	0.5 ± 0.45
	cycle 3	2.5 ± 1.14	0.8 ± 0.52	1.2 ± 1.27	1.3 ± 0.75
	cycle 4	2.5 ± 0.86	0.9 ± 0.31	2.4 ± 0.74	1.2 ± 0.66

Note. The same superscript numbers indicate statistically significant differences in the mean values (Van der Waerden test, P < 0.05). *B – blastocyst stage; M – morula stage; figures indicate gradation of embryos at the blastocyst stage; parentheses indicate the number of embryos that have reached each specific stage.

^**^ Mean values differ from each other in the same way as the average values of the duration of blastomere division periods.

The diversity of development trajectories is notably higher among ME and EM embryos than among EE and MM embryos (see *Fig. 2*; compare the corresponding standard deviations presented in *Table 1*). ME and EM embryos can be subdivided into three groups according to the similarity of trajectories. Average trajectories of development for both ME and EM embryos from these groups differ significantly (*P *= 0.0007 for all comparison variants, paired Wilcoxon test). Embryos of the second group are characterized by a longer duration of the cycle. Durations of the cleavage cycles for embryos of the third group can be comparable or exceed the ones for the first group embryos, and in some cases, for the second group embryos (see details in *Table 2*).

**Table 2:** Time parameters of cleavage cycles (mean value and standard deviation, h) of ME and EM embryos with different terms of development (groups 1, 2 and 3)

		ME			EM		
		Group 1	Group 2	Group 3	Group 1	Group 2	Group 3
Number and prospective stages of embryo development		B5(2), B4(4), B3(1), B1(1)	B2(2), B1(2), M(1)	B5(1), B4(1), B3(1), B1(2), M(1)	B5(5), B4(2)	B1(4), M(1)	B5(3), B4(4), B3(5), B2(4), B1(1)
Zygotic period		25.5 ± 2.01^1,2^	33.3 ± 3.29^1,3^	27.7 ± 2.01^2,3^	25.1 ± 2.19^1,2^	32.9 ± 3.60^1,3^	28.7 ± 2.53^2,3^
Overall cycle duration	cycle 2	11.3 ± 0.62^4,5^	13.3 ± 0.94^4^	13.1 ± 0.87^5^	11.7 ± 1.25^4^	15.0 ± 0.63^4,5^	12.7 ± 1.81^5^
	cycle 3	15.3 ± 3.28^8^	18.4 ± 1.21	21.7 ± 8.22^8^	16.3 ± 1.94^8^	25.7 ± 6.08^8,9^	18.8 ± 3.94^9^
	cycle 4	33.0 ± 3.68^11^	45.5 ± 6.49^11,12^	32.1 ± 7.91^12^	21.9 ± 3.11^13,14^	28.5 ± 4.50^13^	28.5 ± 3.62^14^
Period without divisions	cycle 2	10.5 ± 0.97^6,7^	12.4 ± 0.51^6^	12.2 ± 0.64^7^	10.8 ± 0.41^6^	14.2 ± 0.56^6,7^	11.6 ± 1.21^7^
	cycle 3	11.9 ± 0.87^9,10^	14.6 ± 2.51^9^	13.9 ± 1.08^10^	12.3 ± 1.49^10^	19.6 ± 4.99^10,11^	13.6 ± 2.47^11^
	cycle 4	15.6 ±4.29	21.3 ± 9.22	14.8 ± 7.57	15.6 ± 1.74	15.1 ± 6.65	18.6 ± 4.65
Period of blastomere divisions	cycle 2	0.8 ± 0.60	0.9 ± 0.57	0.93 ± 0.56	0.9 ± 1.01	0.76 ± 0.25	1.08 ± 0.98
	cycle 3	3.4 ± 2.89	3.9 ± 2.18	7.75 ± 7.92	4.0 ± 1.41^12^	6.08 ± 2.16^12^	5.21 ± 3.59
	cycle 4	17.5 ± 2.81	24.2 ± 9.63	17.3 ± 2.66	6.2 ± 2.98^15^	13.4 ± 6.00^15^	9.95 ± 5.19

Notations are the same as in Table 1.

ME embryos of the first, second, and third groups reach the 16-cell stage of development after 85.1 ± 4.28 (mean ± standard deviation), 110.5 ± 6.10, and 94.6 ± 2.57 h; EM embryos – after 74.9 ± 3.10, 102.1 ± 5.20, and 88.8 ± 3.05 h, respectively. The differences in these values are statistically significant (Van der Waerden test, *P *= 0.001, 0.024 and 0.001). At the same time, the average trajectories of the development of ME and EM embryos of the first, second, and third groups do not differ (paired Wilcoxon test; *P *= 0.256, 0.158, and 0.112, respectively).

ME and EM embryos of different groups had reached different stages of development by the end of the registration period (*Table 2*). The first groups included embryos that had formed mature blastocysts (grades 4 and 5). The second groups included slowly developing embryos that had reached the stage of morula or blastocyst that had initiated cavitation. The third groups were diverse since their embryos had a whole spectrum of blastocysts by the end of registration, with blastocysts of grades 2 and 3 being the most presented (*Table 2*). Comparison of alternative distributions (number of embryos that had reached grades 5 and 4 with the number of earlier embryos at the end of the registration period) showed a clear prevalence of both ME and EM embryos in group 1 and, thus, the prevalence of embryos with delayed development in group 2 (two-sided Fisher’s exact test, *P *= 0.021 and 0.001). The same comparison of groups 1 and 3 demonstrated a significant difference in alternative distributions for EM embryos (*P *= 0.019) and similarity among ME embryos (*P *= 0.277).

The period of development for MM embryos to the 16-cell stage occupies an intermediate position between corresponding values for ME and EM embryos of the first groups, and is significantly shorter than the first value (Van der Waerden test, *P *= 0.017) but longer than the second value (*P *= 0.001). The average cleavage trajectory for MM embryos is different from the trajectory for ME and EM embryos of the first groups (paired Wilcoxon test, *P *= 0.001 and 0.000, respectively). Alternative distribution (normally developed embryos compared to embryos with delayed development) in the MM group does not differ from similar distributions in the first groups of ME and EM embryos (Fisher’s exact test, *P *= 0.590 and 0.148) but differs from the distribution in the second groups (*P *= 0.015 for both comparisons).

Averaged parameters of cleavage (and thus the trajectories of development during the cleavage period) for ME and EM embryos of the first groups, as well as for MM embryos (i.e. the parameters of rapidly cleaving embryos in total), are in good agreement with prognostic criteria for successfully developing embryos (see *Table 1* and *Table 2*).

**Blastomere genealogy and cycles**

**Table 3:** Terms and comparison of blastomere cycles in embryos with different variants of the second division of the cleavage

Blastomeres	Mean values ± standard deviation			
	EE (N = 7)	MM (N = 13)	ME(N = 19)	EM (N = 29)
1	11.9 ± 1.29^a^	11.6 ± 1.13^a^	11.7 ± 1.09^a^	11.9 ± 1.46^a^
2	13.1 ± 2.51^a^	12.0 ± 1.09^a^	12.5 ± 1.21^a^	12.9 ± 1.83^a^
1:1	17.6 ± 6.20^b^	14.9 ± 2.76^b,c^	14.6 ± 2.16^b,c^	15.5 ± 4.01^b,c,d^
1:2	21.1 ± 6.15^1,6,b^	16.6 ± 3.20^6,b^	15.5 ± 2.58^1,9,b,d^	18.2 ± 5.34^9,b,e^
2:1	17.7 ± 2.86^2,7,c^	15.0 ± 3.46^7,d^	14.2 ± 2.44^2,10,d,e^	16.3 ± 3.60^10,c,e,f^
2:2	24.4 ± 9.30^3,4,8,c^	16.2 ± 3.47^5,8,c,d^	17.9 ± 5.66^3,c,e^	18.6 ± 4.33^4,5,d,f^
1:1:1	23.6 ± 5.00^d,e,f^	21.8 ± 3.64^15,e,f^	25.3 ± 6.93^f,g^	24.4 ± 5.15^15,g,h,k,l^
1:1:2	29.7 ± 10.71^d^	23.8 ± 3.991^2,16,e,g,h,k^	32.4 ± 9.711^2,f,h,k,l^	28.0 ± 5.891^6,g,m,n,p,q^
1:2:1	22.4 ± 5.36^g^	21.5 ± 4.13^g,l,m,n^	23.0 ± 6.87^h,m,n,p^	24.3 ± 5.32^m,r,s^
1:2:2	32.2 ± 12.50^20,e,g^	22.7 ± 3.67^17,20,l^	28.1 ± 9.74^m^	27.1 ± 5.55^17,h,r,t,u^
2:1:1	25.4 ± 9.17^h^	21.4 ± 3.81^3,18,h,p^	26.0 ± 7.88^13,k,q^	24.5 ± 4.43^18,n,t,v,x^
2:1:2	33.8 ± 11.38^21,22,f,h,k^	24.3 ± 3.67^19,21,f,m,p,q^	30.3 ± 10.76^g,n,q,r^	27.6 ± 4.78^19,22,k,s,v,y,z^
2:2:1	20.2 ± 3.77^k,l^	21.2 ± 3.26^k,q,r^	22.8 ± 6.30^l,r,s^	21.7 ± 5.94^l,p,u,x,y^
2:2:2	26.5 ± 10.22^l^	23.8 ± 3.33^14,n,r^	28.7 ± 6.34^14,p,s^	25.7 ± 5.01^q,z^

Note. The same superscript letters indicate statistically significant differences (P < 0.05, paired Wilcoxon test) in the duration of blastomere cycles in the embryos of each group (value differences are presented in columns). The same superscript numbers indicate statistically significant differences (P < 0.05, Van der Waerden test) of the duration of corresponding cycles in embryos of different groups (value differences are presented in lines).

The duration of blastomere cycles in 4- and 8-cell embryos with different variants of the second cleavage are different in general (Kruskal-Wallis test, *P* < 0.000 in both cases). The shortest cycles are typical for MM embryos; the longest – for EE embryos. ME and EM embryos, the cycles of which for corresponding blastomeres (especially at the 8-cell stage) are sufficiently similar to each other, have an intermediate position (see *Table 3*). The blastomere cycles of the first groups of ME and EM embryos are shorter than the corresponding cycles for embryos of the second and third groups (cycle duration values and their statistical comparisons are presented in *Table 4*).

**Table 4:** The terms of blastomere cycles (mean values, standard deviation, h) and their comparison for ME and EM embryos with different developmental terms (groups 1, 2 and 3)

Blastomeres	ME			EM		
	Group 1 (N=8)	Group 2 (N=5)	Group 3 (N=6)	Group 1 (N=7)	Group 2 (N=5)	Group 3 (N=17)
1	11.0 ± 1.21^1,2,a^	12.4 ± 0.51^1,a^	12.2 ± 0.59^2,a^	10.9 ± 0.46^1,a^	14.2 ± 0.59^1,2,a^	11.7 ± 1.15^2,a^
2	11.7 ± 1.11^3,4,a^	13.3 ± 0.94^3,a^	13.1 ± 0.85^4,a^	11.9 ± 1.33^3,a^	15.0 ± 0.66^3,4,a^	12.7 ± 1.80^4,a^
1:1	13.1 ± 2.10^5,8,b^	16.5 ± 1.77^5^	15.1 ± 0,81^8,b^	13.2 ± 1.90^5,9,b,c^	21.0 ± 6.44^5,10,b^	14.9 ± 2.06^b9,10,b,c^
1:2	13.8 ± 2.43^6,9,b,c^	17.5 ± 2.47^6^	16.1 ± 1.25^9,b^	15.2 ± 2.14^6,b^	25.6 ± 6.95^6,11,b^	17.3 ± 3.76^11,b^
2:1	12.4 ± 0.92^7,10,c,d^	15.5 ± 2.79^7^	15.5 ± 2.24^10,c^	14.2 ± 1.40^7,d^	20.9 ± 4.43^7,12,c^	15.8 ± 2.85^12,d^
2:2	14.9 ± 3.13^13,11,d^	18.2 ± 1.53	21.7 ± 8.24^11,c^	15.8 ± 2.06^8,c,d^	23.0 ± 4.89^8,13,c^	18.4 ± 3.96^13,c,d^
1:1:1	20.0 ± 4.06^12,17,e,f,g^	31.9 ± 5.95^12b,c,d^	26.9 ± 5.45^17,d^	20.4 ± 1.83^14,21,e^	24.3 ± 5.07^d,e^	26.0 ± 5.40^14,21e,f^
1:1:2	27.3 ± 6.78^13,e,h,k^	42.9 ± 9.15^13,20,b,e,f^	30.5 ± 7.15^20,d,e,f^	23.1 ± 3.07^15,22,e,f,g^	31.1 ± 6.18^15,d,f^	29.2 ± 5.72^e22,g,h^
1:2:1	20.1 ± 2.98^h,l^	27.2 ± 11.5^e,g,h^	23.5 ± 4.37^e,g^	19.9 ± 2.79^16,23,h,k^	25.1 ± 3.37^16,g^	25.9 ± 5.69^23,g,k,l^
1:2:2	25.8 ± 7.43^f,l,m^	32.9 ± 14.7^f,g^	27.1 ± 7.63^g^	21.5 ± 3.16^17,24,h,l^	28.3 ± 4.68^17,g^	29.0 ± 5.14^f24,e,k,m,n,p^
2:1:1	19.9 ± 2.62^14,18,k,n,p,q^	34.9 ± 7.18^14,21,k^	26.7 ± 5.70^18,21,h,k^	20.6 ± 3.19^18,25,f,m^	26.8 ± 5.97^18,h,k^	25.4 ± 3.54^25,m,q^
2:1:2	21.9 ± 3.56^15,19,n,r^	42.9 ± 9.99^15,22,c,h,k,l^	30.9 ± 6.89^19,22,h,l^	22.4 ± 2.98^19,26,k,m,n^	28.8 ± 6.31^19,h,l^	29.4 ± 3.34^26,f,l,q,r,s^
2:2:1	23.2 ± 5.00^p,s^	27.8 ± 5.17^23,d,f,l,m^	18.2 ± 6.02^23,f,k,l,m^	19.0 ± 2.20^27,g,l,n,p^	19.4 ± 10.4^3f,k,l,m^	23.6 ± 4.89^27,h,n,r,t^
2:2:2	27.3 ± 5.30^16,g,m,q,r,s^	34.8 ± 4.67^16,24,m^	25.7 ± 6.02^24,m^	21.2 ± 2.92^20,28,p^	28.0 ± 6.86^e20,e,m^	26.8 ± 4.18^28,p,s,t^

Notations are the same as in Table 3.

**Fig. 3:**
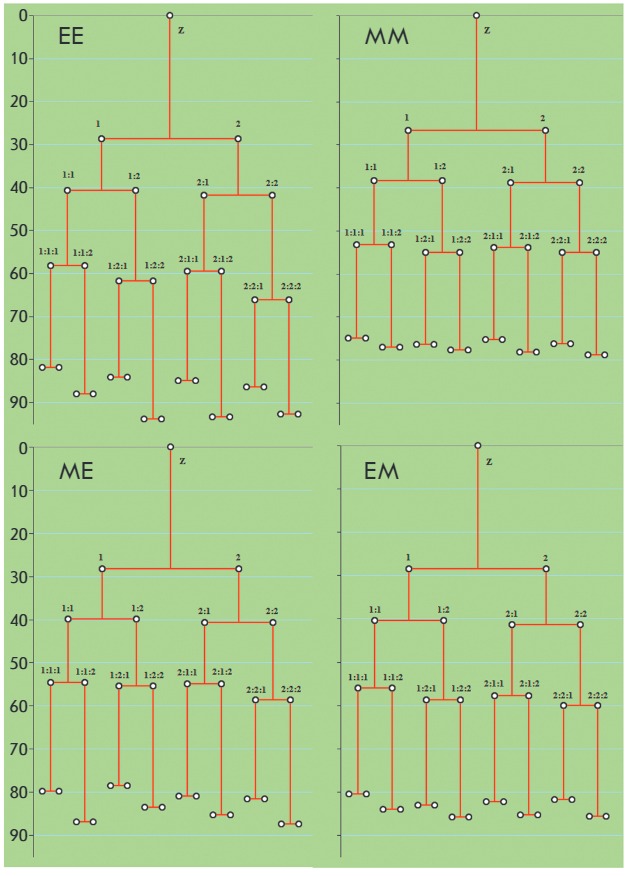
Blastomere genealogy in EE, MM, ME and EM embryos. The numbers at bifurcation points show the order of blastomere appearance (same for every genealogy schemes). The lowest number stands for the shortest cycle length. Vertical axes indicate the time after intracellular sperm injection (h). Z – zygote

Due to the genealogical hierarchy, the division period of any blastomere is composed of a period of dividing blastomere cycle and the periods of the cycles of blastomeres preceding it. Inequality of the cycles of sister blastomeres (*Fig. 3*) is the event that structures blastomere division periods in each cleavage cycle and the trajectories of the whole cleavage.

Substitution of the time parameters of the cleavage trajectory with the sequence (order) of blastomere divisions (that mediate the terms of a blastomere lineage existence, see *Fig. 3*) allows one to identify the diversity of the cycles of individual blastomeres within various embryos. The distributions connecting the division frequencies of blastomeres of similar origin with the sequence of their division in the third and fourth cycles of cleavage trajectories are significantly different from the corresponding equality probabilities for both 4-cell EE, MM, ME, and EM embryos (χ2: 20.0, 40.3, 84.0, and 93.1, respectively; threshold χ2 value (*P * < 0.05) equals 16.09) and 8-cell MM, ME, and EM embryos (χ2: 79.4, 98.9 and 133.8, respectively, threshold χ2 value equals 66.3). The distribution for 8-cell EE embryos does not differ from the equiprobable distribution (χ2 = 56.0,* P *> 0.250), which is due to the small sample size of such embryos.

**Fig. 4:**
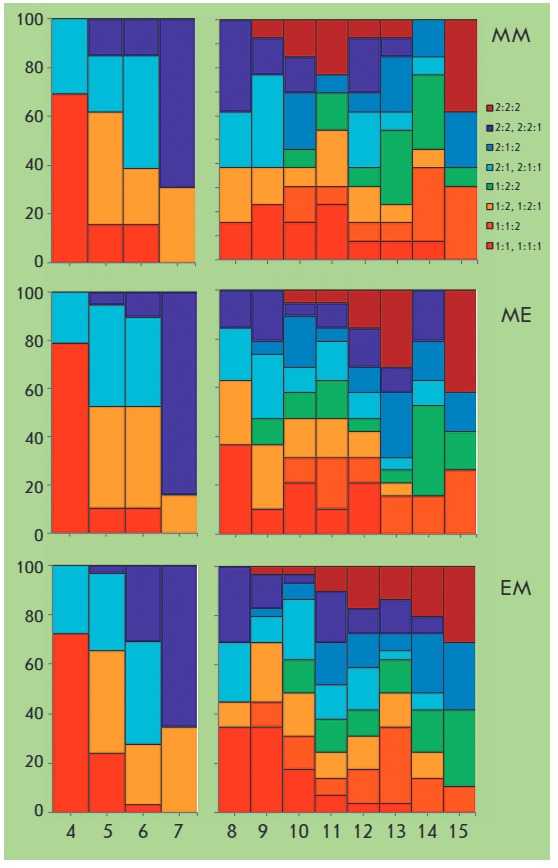
Distribution of blastomere cleavage periods for 4-cell (left diagrams) and 8-cell (right diagrams) aggregates of MM, ME, and EM embryos. Horizontal axes depict the order of successive cellular divisions. Columns of various colors represent the division frequencies of each blastomere in relation to total cell divisions (in %, vertical axes) at a set moment of sequential division. The color code of blastomeres of different origins is presented in the legend in the upper right corner of the diagram

In 4-cell ME embryos, blastomere 1 : 1 divides before the others in 78.9 % of cases (χ2 = 31.7, *P * < 0.000), while the same value for blastomere 2 : 2 is 84.2 % (χ2 = 40.6,* P * < 0.000). Blastomeres 1 : 1 : 1, 2 : 2 : 2, and 1 : 2 : 2 are significantly more likely to divide in the eighth, 15th, and 14th row (36.8, 42.1 and 36.8 %; χ2 = 12.6, 26.1 and 15.1; *P * < 0.050, < 0.001 and 0.050, respectively). Blastomeres 1 : 1 and 2 : 2 in 72.4 and 65.5 % of EM embryos are the fourth and seventh to divide (χ2 = 40.6 and 34.6,* P* < 0.000); blastomere 1 : 1 : 1 is the eighth and ninth to divide (in 34.5 % of cases, χ2 = 36.9 and 22.0, *P * < 0.000 and < 0.005), blastomeres 1 : 1 : 2 and 2 : 2 : 2 are the 14th and 15th (in 31.0 and 34.5 %, χ*2 *= 35.8, *P * < 0.000) (see *Fig. 4*). In MM embryos, the first and the last to divide are blastomeres 1 : 1 and 2 : 2 (69.2 %, χ*2 *= 16.8,* P * < 0.025) and blastomeres 2 : 2 : 1 and 2 : 2 : 2 (in 38.5 % cases, χ*2 *= 15.9 and 18.4, *P * < 0.050 and < 0.025, respectively).

Thus, blastomeres, despite the similarity of origin, significantly differ in their division sequence (i.e. periods of the existence of blastomere lineages until the moment of their division), appear and increase in number during the third and fourth cycles of cleavage in the embryo. The two blastomeres are the first and last to successively divide in the vast majority of 4-cell embryos, while the division sequences of the two other blastomeres vary. In 8-cell embryos, the probability of early or late successive divisions of blastomeres is reduced. However, the number of blastomeres increases; the division moment for them varies among different embryos. If such a tendency is preserved in the fifth and sixth cleavage cycles, the division sequence for all blastomeres, 16- and 32-cell, will become stochastic. Since the embryos, some of them earlier and some of them later, had reached the final stage of development (blastocysts), it can be assumed that the discussed phenomenon is one of the manifestations of the regulatory aspect of blastomeres and embryos in general, which increases during cleavage. An increase in the regulatory aspect, by definition, involves an expansion of the spectrum of potential differentiation pathways. Therefore, one can expect the expansion of potential abilities to differentiate in blastomeres of 8-cell and, particularly, 16- and 32-cell embryos.

It is traditionally believed that oocytes and zygotes are totipotent. However, according to cytologic criteria, oocytes and zygotes inheriting the structure of oocyte are highly specialized cells. In addition to their characteristic morphology and specific syntheses, zygote specialization is manifested in polarization with uneven volumetric distribution (as well as in the length of the cortical layer) of cellular organelles and the complex of specific regulatory macromolecules [18-20 et al.]. We suggest that blastomeres acquire the ability of self-synthesis and regulation, polarization, and formation of functional contacts and, finally, of specific differentiation only after the elimination of the specific characteristics of zygote organization and release from the influence of zygotic determinants [21-23]. At the molecular genetic level, the events that release blastomeres from “zygote dictatorship” during the period of the first cleavage divisions are poorly studied and are most likely associated with cell cycle regulation [24, 25].

## CONCLUSION

In placental mammals, unlike in lower vertebrates, polarization (pre-mapping) of zygote is sufficiently labile, which determines the diversity of time parameters in the early development and, in the case of imperfection or insufficiency of this pre-mapping, a rather high level of early development anomalies. As a result of the realization of all possible combinations of meridional and equatorial furrows, four variants of 4-cell embryos are formed during the cleavage of 2-cell human embryos (as well as mouse embryos and, probably, other types of placental mammals). The blastomeres of embryos that belong to different variants include significantly different parts of zygotes, thus acquiring different “doses” of determinants. Segregation of zygotic cytoplasm and determinants continues during further cleavage divisions. This, in turn, is reflected in the significant diversity in time parameters (blastomere cycles, cleavage cycles and cleavage trajectory in general) in the next cleavage rounds of each variant of 4-cell embryos. The diversity associated with the degree of “perfection” of zygote organization interferes with the diversity provided by way of its segregation, which is manifested in cleavage trajectories. An example of this is the development trajectories for ME and EM embryos with significantly different cleavage rates (the first, second, and third groups). According to preliminary estimates, the formation frequencies of implantation-competent blastocysts tracing to each of the variants of 4-cell embryos are different. Thus, the variant of a 4-cell embryo formation should be taken into account during early prognosis of the prospectivity of each individual embryo.

Substitution of time parameters by ordinal characteristics (i.e. the sequences of blastomere divisions in the cleavage trajectory or, in other words, mediated terms of the existence of blastomeres lineages) allows one to reveal another form of diversity associated with the characteristics of the cleavage process itself – the diversity in the moment of entry into the next cleavage cycle for blastomeres of similar origin within various embryos. We suggest that this phenomenon may be explained by the gradual decrease in the effects of determinants as a reflection for consecutive elimination of the specific organization of zygote in its fragments (i.e. blastomeres). A dedifferentiation of blastomeres achieved in this way precedes and, possibly, enables their own expression, regulation, and, finally, their own differentiation. Greater or lesser success in blastomeres’ dedifferentiation defines a time shift (longer or shorter, respectively) for the events of asymmetric divisions and compaction, which in turn may influence the ratio of inner cell mass to mural trophectoderm in blastocysts.
